# Endophytic Microbial Consortia of Phytohormones-Producing Fungus *Paecilomyces formosus* LHL10 and Bacteria *Sphingomonas* sp. LK11 to *Glycine max* L. Regulates Physio-hormonal Changes to Attenuate Aluminum and Zinc Stresses

**DOI:** 10.3389/fpls.2018.01273

**Published:** 2018-09-04

**Authors:** Saqib Bilal, Raheem Shahzad, Abdul L. Khan, Sang-Mo Kang, Qari M. Imran, Ahmed Al-Harrasi, Byung-Wook Yun, In-Jung Lee

**Affiliations:** ^1^School of Applied Biosciences, Kyungpook National University, Daegu, South Korea; ^2^Natural and Medical Sciences Research Center, University of Nizwa, Nizwa, Oman

**Keywords:** soybean, endophytes, macronutrients, soil extracellular enzymes, hormones, reactive oxygen species, antioxidants, heavy metal ATPases

## Abstract

The compatible microbial consortia containing fungal and bacterial symbionts acting synergistically are applied to improve plant growth and eco-physiological responses in extreme crop growth conditions. However, the interactive effects of phytohormones-producing endophytic fungal and bacterial symbionts plant growth and stress tolerance under heavy metal stress have been least known. In the current study, the phytohormones-producing endophytic *Paecilomyces formosus* LHL10 and *Sphingomonas* sp. LK11 revealed potent growth and tolerance during their initial screening against combined Al and Zn (2.5 mM each) stress. This was followed with their co-inoculation in the Al- and Zn-stressed *Glycine max* L. plants, showing significantly higher plant growth attributes (shoot/root length, fresh/dry weight, and chlorophyll content) than the plants solely inoculated with LHL10 or LK11 and the non-inoculated (control) plants under metal stresses. Interestingly, under metal stress, the consortia exhibited lower metal uptake and inhibited metal transport in roots. Metal-induced oxidative stresses were modulated in co-inoculated plants through reduced hydrogen peroxide, lipid peroxidation, and antioxidant enzymes (catalase and superoxide dismutase) in comparison to the non-inoculated plants. In addition, endophytic co-inoculation enhanced plant macronutrient uptake (P, K, S, and N) and modulated soil enzymatic activities under stress conditions. It significantly downregulated the expression of heavy metal ATPase genes *GmHMA13, GmHMA18, GmHMA19*, and *GmPHA1* and upregulated the expression of an ariadne-like ubiquitin ligase gene *GmARI1* under heavy metals stress. Furthermore, the endogenous phytohormonal contents of co-inoculated plants revealed significantly enhanced gibberellins and reduced abscisic acid and jasmonic acid contents, suggesting that this endophytic interaction mitigated the adverse effect of metal stresses in host plants. In conclusion, the co-inoculation of the endophytic fungus LHL10 and bacteria LK11 actively contributed to the tripartite mutualistic symbiosis in *G. max* under heavy metal stresses; this could be used an excellent strategy for sustainable agriculture in the heavy metal-contaminated fields.

## Introduction

Plants, being sessile in nature, are often exposed to adverse environmental perturbations, which significantly hamper their growth and development, resulting in significant yield loss. Of those environmental stresses, the excessive release of heavy metals and their accumulation in agricultural soils due to continuous technological advancement in industrialization and urbanization has become a serious global concern, which not only impairs crop productivity and soil quality, but also poses serious threats to human health upon accumulation in agricultural products (Khan et al., 2015; Ma et al., 2016a). The serious threats of heavy metal contamination to plant growth and human health necessitate the development of efficient and eco-friendly soil remediating techniques.

Recently, plant–microbe interactions under metal toxicity have gained considerable attention for remediating contaminated soil and improving plant physiology. Various studies have demonstrated the substantial effect of microbial (bacterial and fungal) inoculation on alleviating heavy metal stress as well as on boosting plant growth, development, and nutritive status under metal-contaminated soil conditions. Such synergistic effects of microbes are accredited to the production of siderophores, phytohormones, such as gibberellins, auxin, ethylene, and enzymes, such as ACC-deaminase, which provide an extra arsenal to overcome the adverse effects of metal toxicity. A number of studies have illustrated the impact of the single inoculation of biotic abiotic stress mitigation including metal-resistant and plant growth-promoting microorganisms, including rhizobacteria *Pseudomonas libanensis, Pseudomonas reactans, Micrococcus luteus* (Ma et al., 2016b; Pinter et al., 2017) endophytic *Pseudomonas azotoformans, Streptomyces* sp. (Ma et al., 2017; Qin et al., 2017), arbuscular mycorrhizal (AM) fungi *Rhizophagus irregularis, Funneliformis mosseae*, and *F. caledonium* (Mnasri et al., 2017; Zhang et al., 2018), and endophytic *Thermomyces* sp, *Piriformospora indica, Penicillium janthinellum* (Shahabivand et al., 2017; Ali et al., 2018) on alleviating environmental stresses including the metal toxicity-induced stresses and improving soil remediation efficiencies. Conversely, the impact of the co-inoculation of bacterial and fungal interaction on plants under abiotic stresses, including metal toxicity, is rarely studied. The impacts of the synergistic interaction between fungi and bacteria are reported to be beneficial for plant growth promotion by Liu et al. (2012), as the survivability, growth, and development of *Verbascum lychnitis* under Zn and Pb substrates were significantly improved by co-inoculating it with endophytic and AM fungi (Wężowicz et al., 2017). Similarly, the positive effects of reduced excess zinc toxicity and modulated micronutrient uptake in *Glycine max* L. have been observed with the combined inoculation of AM *Gigaspora rosea* and rhizobacterium *Bradyrhizobium diazoefficiens* by Ibiang et al. (2017). The positive outcomes produced by the interaction of bacteria and fungi on plant growth stimulation and regulation of stress tolerance under hostile conditions, including heavy metal stresses, have been extensively reviewed by Martin et al. (2017) and Rozpądek et al. (2017).

In spite of such beneficial consortium, studies investigating the association of bacteria and fungi, particularly the specific interaction between endophytes, in hostile environment are limited. Endophytes (bacteria and fungi) have been extensively reported for their beneficial association with plants for their growth and development, fitness, diversification and mitigation of stresses, including metal-induced toxicity, under harsh environmental conditions (Hardoim et al., 2015). Therefore, their manipulation in the metal-contaminated soil for promoting plant growth and reducing metal toxicity will demonstrate a better knowledge of endophytes synergism and development of appropriate phytoremediation practices. As the persistence of heavy metal toxicity in soil leads to the significant impairment of normal plant functions in a variety of ways, including the impediment of different metabolic processes by the destruction of enzymatic activities, severe disruption of cytoplasmic membrane integrity, excessive production of reactive oxygen species (ROS) (Emamverdian et al., 2015).

Aluminum (Al) is one of the most abundant heavy metals in the earth’s crust and is also one of the major inhibitors of crop production and growth. It exhibits toxicity in the acidic soil (pH < 5.0 or 5.5), where it turns into its most phytotoxic form (Al^3+^) (Saby et al., 2017). No beneficial biological role of Al has been proven in plants; however, it primarily adversely affects the root growth and consequently leads to the deterioration of the aerial plant parts due to early root damage (Xu et al., 2017). Furthermore, it hampers nutritional uptake and impairs cell wall, plasma membrane, cytoskeleton, and nucleus at the cellular level (Emamverdian et al., 2015). Similarly, zinc (Zn) is considered an essential metal, but its excessive levels in the agricultural soil cause stress in plants, leading to the reduced root and shoot growth, curling of young leaves, necrotic spots in mature leaves, leaf chlorosis, reduced photosynthesis, as well as excessive generation of ROS, leading to adverse effects on membrane integrity and permeability, etc. (Shi et al., 2015). Excessive accumulation of metals, including Al and Zn, has also been reported to adversely degrade the activity of metal transporter family members, including H^+^-ATPase, low-affinity cation transporters, and ABC transporters (Singh et al., 2016). In addition, Al and Zn adversely affect the soil extracellular enzymatic activity. Wallenstein and Weintraub (2008) and Ali et al. (2017) have reported the adverse influence of Zn and Al toxicity on soil extracellular enzymatic activity.

Soybean is an important edible crop and a vital source of vegetable oil and proteins worldwide. The growth, yield, and quality of soybean can be significantly damaged by a number of adverse environmental stresses, including metal toxicity. Therefore, the current study was aimed to investigate the co-inoculation effects of the synergistic consortium of the phytohormones Indole-3-acetic acid and gibberellins (IAA and GAs)-producing (Khan et al., 2012, 2014) compatible endophytic bacteria *Sphingomonas* sp. LK11 and fungus *Paecilomyces formosus* LHL10. We aimed to comprehensively assess the metal detoxification, stress alleviation, and growth promotion in *G. max* under Zn- and Al-contaminated soil conditions to investigate the influence of endophytes co-inoculation. Here, we described plant biomass regulation, photosynthesis performance, hormonal regulation and antioxidative system, heavy metal accumulation, heavy metal transporter-mediated translocation, and nutrient acquisition, as well as an improvement in soil extracellular enzymatic activity caused by the co-inoculation of endophytic LK11 and LHL10. The ultimate goal of this study was to develop an ecofriendly phytoremediation strategy for efficiently coping with Al- and Zn-contaminated soil.

## Materials and Methods

### Screening of Endophytic LHL10 and LK11 Tolerance Against Heavy Metals

In the current study, the previously isolated endophytic *Paecilomyces formosus* LHL10 (HQ444388) and *Sphingomonas* sp. LK11 (KF515708) were selected on the basis of their abilities to produce phytohormones, gibberellins (GAs) and Indole-3-acetic acid (IAA) and screened out against different concentrations of Al and Zn (1.5, 2.5, and 3.5 mM each) in potato dextrose broth to assess their metal-tolerating abilities for 7 days and 72 h, respectively. Of all screening concentrations, the media containing 2.5 mM each of Al and Zn showed the best growth of both LHL10 and LK11. Therefore, the fungal mycelia and bacterial cells were harvested via centrifugation from the media containing 2.5 mM of each metal and subjected to the inductively coupled plasma-mass spectrometry (ICP-MS) in order to evaluate their metal-uptake and remediating potential.

### Host–Plant and Endophyte Interaction Under Al and Zn Stress

The *G. max* seeds were surface sterilized for 30 s with 70% ethanol followed by a treatment with 5% NaOCl for 15 min and then rinsed with autoclaved double-distilled water. The soybean seeds were sown in autoclaved pots containing 150 g of horticulture soil composed of cocopeat (68%), perlite (11%), zeolite (8%), as well as micronutrients available as NH^4+^ ∼0.09 mg g^-1^; P_2_O_5_ ∼0.35 mg g^-1^; NO_3-_ ∼0.205 mg g^-1^; and K_2_O ∼0.1 mg g ^-1^ and kept in a growth chamber (day/night cycle: 14 h; 28°C/10 h; 24°C; relative humidity 60–70%; light intensity 1000 E m^-2^ s^-1^ Natrium lamps). In case of sole inoculation, the metal-resistant endophytic strains LHL10 (1–2 g of mycelia) and LK11 (10^8^ CFU mL^-1^) were separately inoculated in the rhizoshperic area of the 12-day-old soybean seedlings, while in case of dual inoculation, both endophytes LHL10 and LK11 were simultaneously applied to the rhizoshperic area of the seedlings to establish a symbiotic association between endophytes and plants. Subsequently, 80 mL of a solution containing Al and Zn (2.5 mM each) was applied to the 18-day-old seedlings for 12 days. Thereafter, the treated plants were harvested, frozen in liquid nitrogen, and transferred to -80 °C for further biochemical analysis. The growth attributes of the plants under each treatment were recorded and their chlorophyll contents were measured by a chlorophyll meter (SPAD-502 Minolta, Japan). The experiment was replicated three times, and each replicate included 20 plants. The experimental design included the following treatments: (i) control plants treated with only water, (ii) sole endophytic LHL10-treated plants without Al and Zn stress, (iii) sole endophytic LK11-treated plants without Al and Zn stress, (iv) LHL10 and LK11 co-inoculated plants without metal stress, (v) only Al- and Zn-treated plants, (vi) endophytic LHL10-treated plants with Al and Zn stress, (vii) endophytic LK11-treated plants with Al and Zn stress, (viii) LHL10- and LK11-co-inoculated plants with metal stress. The experiment was performed three times, with 20 replicates per treatment.

### Analysis of Heavy Metal (Al and Zn) and Macronutrient Uptake in Plant Roots and Shoots via ICP-MS

The freeze-dried powdered samples were used for the quantification of metals (Al and Zn) and nutrient (Potassium, K; Phosphorus, P; Calcium, Ca; and Sulfur, S) uptake in roots and shoots as well as in microbial cells using ICP-MS (Optima 7900DV, Perkin-Elmer, United States), while Nitrogen (N) was quantified with an elemental analyser (Flash2000; ThermoFisher Scientific, Waltham, MA, United States). The translocation efficiency was assessed by measuring the translocation factor (TF), biological concentration factor (BCF), and biological accumulation factor (BAF) as reported by Waqas et al. (2014) and Bilal et al. (2017).

### cDNA Synthesis and qRT-PCR

Total RNA was extracted from the soybean roots and leaves by the protocol adopted by Imran et al. (2018). Briefly, the roots and leaves (1.0 g) were grounded to a fine powder in liquid nitrogen using a chilled mortar and pestle and immediately transferred to the RNA-free conical centrifuge tubes (Falcon), followed by the addition of the TRIzol reagent (Invitrogen, Carlsbad, CA, United States) and centrifugation (13000 rpm) for 10 min at 4°C. Thereafter, 200 μL of chloroform was added to the supernatants, vortexed vigorously for 15 s, and incubated on ice for 3 min, followed by centrifugation (13000 rpm) for 15 min at 4°C. Subsequently, the aqueous phase was transferred to a fresh centrifuge tube containing 50 μL of isopropanol followed by centrifugation (13000 rpm) at 4°C. The supernatant was discarded carefully, and the pellet was washed with 75% ethanol. All samples were dried and resuspended in nuclease-free water, and the RNA concentration was measured using NanoQ (OPTIZEN Korea). The cDNA synthesis and quantitative PCR (qPCR) were performed as described previously by Imran et al. (2018). Briefly, 2 μg of RNA was used to synthesize cDNA using the BioFACT^TM^ RT-Kit (BIOFACT, Korea) according to the manufacturer’s standard protocol. The synthesized cDNA was used as a template in a two-step qRT-PCR reaction conducted to quantify transcript accumulation using an Illumina Eco^TM^ system (Illumina, United States). The reaction mixture was prepared by adding 10 μL of 2X Real-Time PCR master mix, including SYBR^®^ Green I (BioFACT, Korea) and 1 μL of each primer (a total of 10 primers) for the respective genes studied. The PCR conditions were initial activation at 95°C for 15 min, followed by 40 cycles of denaturation at 95°C for 20 s and annealing at 60°C for 40 s. The melting curves were analyzed to verify the amplicon specificity of each primer pair. The expression of each gene was measured relative to the expression of the internal control gene *Actin*. **Supplementary Table S1** shows the list of genes and their specific primers.

### Soil Extracellular Enzyme Analysis

To quantify the soil extracellular enzymes, the method of Khan et al. (2016) was adopted with some modifications. Briefly, the soil (1 g/50 mL acetate buffer (50 mM; pH 5.5) samples from all treatments were incubated at 26°C and shaken at 150 rpm. Each sample had five replicates. After 24 h, a clear supernatant was obtained by centrifuging (12,000 rpm at 4°C for 10 min) the solution in each flask. The known standard (4-methylumbelliferone) and respective florigenic substrates for each enzyme were prepared according to Khan et al. (2016). The pre-optimized fluorescence spectrophotometer (Shimadzu, Tokyo, Japan) was used to read the absorbance at 360-nm excitation and 460-nm emission at time initial and 30-min intervals for 2 h. The readings were calculated according to the formula: Activity (μmol h^-1^ L^-1^) = slope of concentration versus time in hours.

### Quantification of Hydrogen Peroxide, Lipid Peroxidation, and Antioxidants

The measurement of the H_2_O_2_ concentration in soybean leaves was carried out by the method described by Li et al. (2015). Briefly, 300 mg of plant leaf samples were homogenized with 3 mL of 50 mM phosphate buffer (pH 6.5). The homogenates were centrifuged at 6000 rpm for 25 min. Subsequently, 3 mL of the supernatant was mixed with 1 mL of 0.1% titanium dioxide in 20% H_2_SO_4_ (w/v) and again centrifuged at 6000 rpm for 15 min. The absorbance of the yellow color of supernatant was read at 410 nm. The H_2_O_2_ content was calculated by using a standard curve with known concentrations and expressed as μM g^-1^ FW. The lipid peroxidation levels of soybean leaves were measured spectrophotometrically as described by Bilal et al. (2017). The concentration of lipid peroxidation products was expressed as malondialdehyde (MDA) formed per gram tissue weight.

Similarly, the activities of enzymatic antioxidants, including catalase (CAT) and superoxide dismutase (SOD), in soybean tissues were measured as described by Sirhindi et al. (2016), and their absorbances were measured via spectrophotometer at 240 and 540 nm, respectively and expressed as U/mg protein. One SOD unit is the quantity of enzyme that inhibits 50% photo reduction of nitroblue tetrazolium (NBT).

### Extraction and Quantification of Soybean Phytohormones—Gibberellins (GAs), Abscisic Acid (ABA), and Jasmonic Acid (JA)

The extraction and quantification of the soybean GAs were carried out according to Hamayun et al. (2017), and the extract was subjected to chromatography and mass spectroscopy for GA detection, identification, and quantification. The seedlings were crushed in liquid nitrogen to a fine powder. The GAs were extracted and quantified by using 0.5 g of the fine powder, which was supplemented with [^2^H_2_] GA internal standards. The instrument used for the determination of GA was a gas chromatograph (Hewlett-Packard 6890, 5973N mass selective detector). The gas chromatography-mass spectroscopy coupled with selected ion monitor (GC/MS-SIM) conditions used for the analysis and quantification of GAs (GA1, 4, 9, and 24) are listed in **Supplementary Table S2**.

The endogenous ABA was extracted from the plants according to the protocol described by Qi et al. (1998), with slight modifications as reported by Shahzad et al. (2017). Briefly, the freeze-dried plant samples were used for extraction and chromatography with an internal standard Me-[^2^H_6_]-ABA. The resultant extract was dried using the N_2_ gas followed by methylation with diazomethane for the detection and quantification of ABA by GC–MS coupled with SIM (5973 Network Mass Selective Detector and 6890 N Network Gas Chromatograph, Agilent Technologies, Santa Clara, CA, United States). The Lab-Base (ThermoQuset, Manchester, United Kingdom) data system software was used to monitor the signal ions at *m*/*z* 162 and 190 for Me-ABA and at *m*/*z* 166 and 194 for Me-[^2^H_6_]-ABA. The experiment was replicated three times. The GC/MS-SIM conditions used for the analysis and quantification of ABA are provided in **Supplementary Table S3**.

To measure the level of JA in soybean, 0.4 g of freeze-dried powder was used as reported by Hamayun et al. (2017). Briefly, the freeze-dried sample was extracted by 50 mM citric acid and acetone (30:70, v/v) having 30 ng of [9, 10-^2^H_2_] Dihydro-JA as an internal standard. The extracts were analyzed by GC-MS (6890 N Network GC System and 5973 Network Mass Selective Detector; Agilent Technologies). We monitored the fragment ion at *m/z* 1/4 83 amu, corresponding to the base peaks of JA and [9, 10-^2^H_2_] Dihydro-JA. The GC/MS-SIM conditions used for the analysis and quantification of JA are mentioned in **Supplementary Table S4**.

### Statistical Analysis

All experiments were performed independently four to five times. All data were subjected to the analysis of variance (ANOVA) followed by Duncan’s multiple range test using SAS version 9.2 (Cary, NC, United States), and the data displayed are the mean values with standard error (SE).

## Results

### Effect of Endophytic Co-inoculation on Plant Growth-Promoting Attributes Under Al and Zn Stress

**FIGURE 1:**
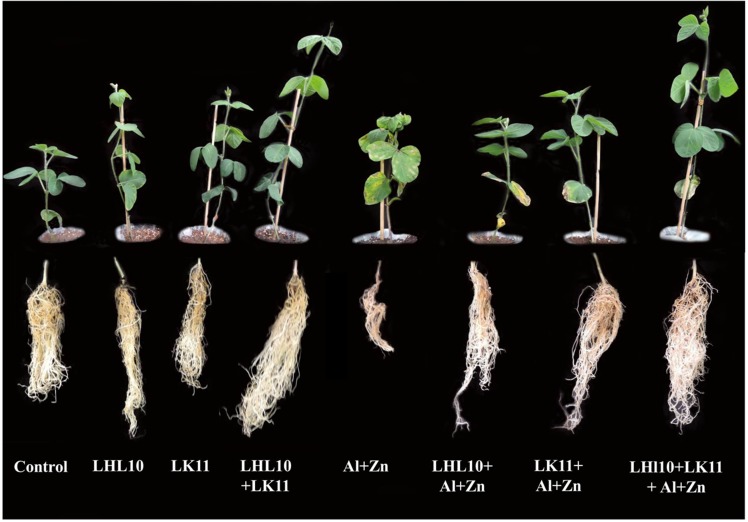
Influence of the co-inoculation of endophytic *Paecilomyces formosus* LHL10 and *Sphingomonas* sp. LK11 on soybean growth under metal (Al/Zn) stress. Each plant represents 20 replicates of eight treatments.

Screening of endophytic LHL10 and LK11 tolerance against heavy metals displayed that LHL10 mycelia and LK11 bacterial cells can approximately accumulate 83 and 79% Zn and 77 and 73% Al, respectively. Thereafter, the application of co-inoculation on soybean growth attributes disclosed the significant effects of the endophytic co-inoculation and sole inoculation on the growth-promoting attributes of the soybean plants compared to the non-inoculated plants (**Figure 1**). In the absence of stress, the endophytic co-inoculation significantly enhanced the shoot length by 71.3, 34.11, and 46.3% and the root length by 90.7, 27.1, and 34.8% when compared with the root and shoot lengths of the non-inoculated, sole LHL10-inoculated, and sole LK11-inoculated plants, respectively (**Table 1**). The fresh and dry weights increased significantly by 71.8 and 130.1% in the co-inoculated plants, followed by 38.9 and 70.5% in the sole LHL10-inoculated plants, and by 10.6 and 52.2% in the sole LK11-inoculated plants, respectively, compared to the fresh and dry weights of the non-inoculated plants under control conditions. However, the metal stress substantially retarded the growth attributes of the non-inoculated plants; nevertheless, the endophytic co-inoculation significantly mitigated the adverse effects of Al and Zn toxicity by displaying significantly higher growth attributes in co-inoculated plants compared to the non-inoculated and sole-inoculated plants. The metal toxicity significantly reduced the shoot and root lengths by 1.8 and 1.7 times in non-inoculated plants, followed by nearly 1.5 and 1.2 times in the LK11-inoculated plants, and by approximately 1.2 and 1.07 times in the LHL10-inoculated plants, respectively, compared to the root and shoot lengths of the co-inoculated plants. On exposure to metals stress, the co-inoculated plants displayed significant restoration of the fresh and dry biomass to the level of control conditions of such plants. The co-inoculated plants displayed significant fresh biomass under stress conditions followed by the LHL10-inoculated, LK11-inoculated, and non-inoculated plants. Same trend line was observed in case of the dry biomass of the co-inoculated plants under stress conditions, but the sole-inoculated plants showed insignificant differences with each other. A significant increase (*p <* 0.005) was detected in the chlorophyll content of the co-inoculated plants under control as well as stress conditions compared to the sole-inoculated as well as non-inoculated plants.

**Table 1:** Effect of endophytes inoculation on soybean growth under Al/Zn stress.

Treatment	S.L (Cm)	R.L (Cm)	S.F.W. (g)	S.D.W (Cm)	CC (SPAD)
**Without metals stress**					
Control	23.4 1.70^c^	11.2 1.17^c^	13.7 0.73^d^	5.7 0.34^c^	37.6 0.91^c^
LHL10	29.9 2.38^b^	14.1 1.28^b^	19.1 0.57^b^	9.8 0.59^b^	44.13 0.81^b^
LK11	27.4 1.53^b^	13.3 0.98^b^	15.2 0.57^c^	9.0 0.36^b^	43.51 0.87^b^
LHL10 + LK11	40.1 2.10^a^	17.9 0.76^a^	23.6 0.75^a^	13.3 0.55^a^	48.13 0.95^a^
**With metals stress**					
Control + Metals	17.0 1.80^d^	9.7 0.51^c^	9.4 0.61^d^	4.8 0.40^c^	16.53 1.51^d^
LHL10 + Metals	27.1 1.56^b^	16.1 0.46^a^	13.3 0.65^b^	7.3 0.27^b^	29.21 0.45^c^
LK11 + Metals	21.5 1.99^c^	14.7 0.83^b^	12.0 0.37^c^	7.7 0.33^b^	34.03 1.23^b^
LHL10 + LK11 + Metals	32.0 2.91^a^	17.2 0.70^a^	20.4 0.58^c^	12.0 0.57^a^	41.33.2 1.02^d^


*SL, shoot length; RL, root length; SFW, seedlings fresh weight; SDW, seedlings dry weight; CC, chlorophyll content. Each value represents mean ± SD of 20 replicates from three independent experiments. Values with different letters in a column are significantly different at the 5 % level of probability by DMRT.*

### Co-inoculation Influences Al and Zn Uptake in *Glycine max* L. and Availability in Soil

The influence of endophytes co-inoculation on metal uptake and translocation was tested in plant roots and shoots to investigate the plant-endophyte interaction efficiency in remediating the combined toxicity of Al and Zn (**Table 2**). Under control conditions, the inoculated as well as non-inoculated plants either did not exhibit or posed intangible levels of Al and Zn in their roots and shoots. However, spiking the soil with combined Al and Zn metals under stress treatment significantly enhanced the root and shoot uptake of Al and Zn in all treatments, resulting in poor growth compared to the control conditions. The accumulation of Al ions by the roots and their translocation to the shoots were detected to be substantially higher as compared to the Zn ions. The introduction of endophytes remarkably decreased the uptake and translocation of Al and Zn by several folds in the co-inoculated plants compared to the non-inoculated plants. The endophytic co-inoculation was found to be the most efficient (*p <* 0.005) for lowering Al-ion uptake by roots and translocation to shoots, followed by the sole LK11 inoculation, sole LHL10 inoculation, and no inoculation (control) conditions. Similarly, the co-inoculated plants substantially reduced the levels of Zn uptake and translocation by 299.1%, followed by the LHL10- and LK11-inoculated plants, which showed 223.1 and 176.4% reduction in roots and 326.7, 170.9, and 266.8% reduction in shoots compared to the non-inoculated plants. The influence of the endophytic co-inoculation on plant Al and Zn uptake and translocation was further scrutinized by calculating the BCF, BAF, and TF values (**Table 2**). In the absence of heavy metal stress, the introduction of co-inoculation led to a relatively high BCF in case of Al, whereas in case of Zn, all treatments showed a similar and negligible BCF in the absence of metals stress. Under metals stress, the sole LK11-inoculated plants revealed higher BCF in case of both Al and Zn, while LHl-10 showed the least BCF. The BAF under metals stress remained high in the non-inoculated plants in case of both Al and Zn, i.e., 0.52 and 0.67, respectively. In the absence of heavy metal stress, the sole LK11-inoculated and non-inoculated plants led to an increase in the TF in case of both Al and Zn stress conditions. Under metal stress, the TF was almost similar and ranged between 0.5 to 0.7 in case of Al and 0.7 to 0.8 in case of Zn. Furthermore, we explored the influence of co-inoculation on the availability and remediation of Al and Zn in soil (**Table 2**). The results showed that the sole-inoculated as well as co-inoculated plants markedly immobilized Zn and Al compared to the non-inoculated plants under heavy metal stress. The soil Zn was detected to be markedly immobilized (118.3 ± 5.2 mg kg^-1^ D.WT) by co-inoculation, which was equally followed by the sole inoculation of LHL10 and LK11 under heavy metal stress. Likewise, the application of endophytic co-inoculation led to the significant immobilization of Al (243.7 ± 9.7 mg kg^-1^ D.WT) in soil by displaying approximately 2.0-, 1.5-, and 1.2-fold less availability in comparison with the sole LHL10-inoculation, sole LK11-inoculation, and no inoculation (control) treatments.

**Table 2:** Al and Zn uptake, compartmentalization in *Glycine max* L. and their remediation in soil with or without endophytes interaction.

Treatment	Shoot Al (mgkg^-1^)	Root Al (mgkg^-1^)	Soil Al (mgkg^-1^)	Shoot Zn (mgkg^-1^)	Root Zn (mgkg^-1^)	Soil Zn (mgkg^-1^)	BCF (Al)	BAF (Al)	TF (Al)	BCF (Zn)	BAF (Zn)	TF (Zn)
**Without metals stress**
Control	ND	ND	ND	0.015 ± 0.003^a^	0.019 ± 0.002^b^	0.33 ± 0.05^b^	ND	ND	–	0.05	0.04	0.78
LHL10	ND	ND	ND	0.012 ± 0.004^a^	0.042 ± 0.003^a^	0.39 ± 0.02^b^	ND	ND	–	0.10	0.03	0.28
LK11	0.11 ± 0.03^a^	0.14 ± 0.02^b^	0.28 ± 0.03^a^	0.013 ± 0.005^a^	0.021 ± 0.003	0.52 ± 0.04^a^	0.51	0.39	0.78	0.04	0.02	0.61
LHL10 + LK11	0.09 ± 0.002^a^	0.22 ± 0.04^a^	0.23 ± 0.04^a^	ND	ND	0.58 ± 0.03^a^	0.94	0.35	0.40	0.00	0.00	–
**With metals stress**												
Control + Metals	241.2 ± 14.2^a^	325.2 ± 18.4^a^	479.3 ± 24.7^a^	266.2 ± 17.4^a^	376.4 ± 27.6^a^	392.3 ± 2.1^a^	0.67	0.52	0.74	0.95	0.67	0.70
LHL10 + Metals	125.3 ± 7.83^c^	201.5 ± 12.6^c^	359.3 ± 27.4^b^	82.4 ± 5.2^c^	102.6 ± 4.5^c^	168.5 ± 6.3^b^	0.56	0.34	0.62	0.60	0.48	0.80
LK11 + Metals	148.8 ± 6.58^b^	276.3 ± 16.5^b^	302.6 ± 17.6^c^	96.3 ± 6.7^b^	138.9 ± 13.7^b^	155.2 ± 5.7^b^	0.91	0.49	0.53	0.89	0.62	0.69
LHL10 + LK11 + Metals	92.2 ± 5.75^d^	170.4 ± 7.6^d^	243.7 ± 9.74^d^	66.7 ± 5.8^d^	88.2 ± 5.2^d^	118.37 ± 7.9^c^	0.69	0.37	0.54	0.74	0.56	0.75


*BCF, BAF, TF denotes biological concentration factor, biological accumulation factor and translocation factor of Al and Zn respectively. Data of columns indexed by the different letter within each treatment are significantly different at the 5% level (*p* < 0.05) of probability by DMRT.*

### Effect of Endophytic Co-inoculation on the Transcript Levels of Different Metal Transporter Genes

**FIGURE 2:**
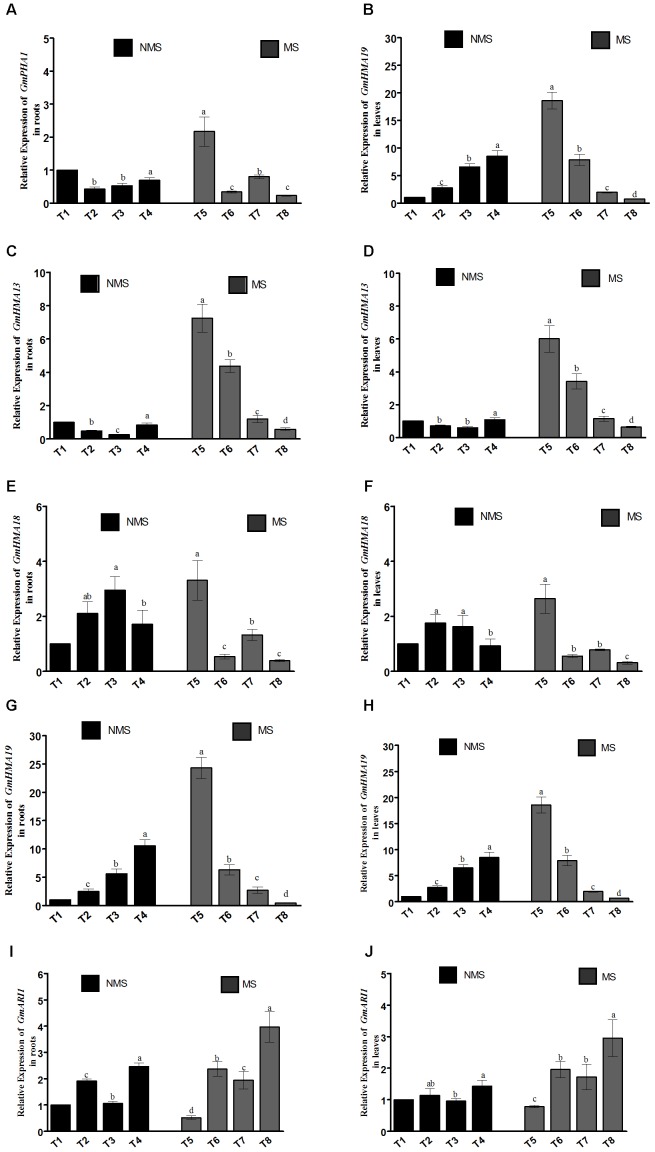
Influence of the endophytic inoculation on the expression patterns of **(A,B)** *GmPHA1* **(C,D)** *GmHMA13*, **(E,F)** *GmHMA18*, **(G,H)***, GmHMA19*, and **(I,J)** *GmARI1* in soybean roots and leaves under Al and Zn stress. NMS represents “No metal stress” while MS represents “Metal stress.” T1 (Control Plants), T2 (LHL10-treated plants), T3 (LK11-treated plants), T4 (LHL10 + LK11-treated plants), T5 (Control + Metals), T6 (LHL10 + Metal-treated plants), T7 (LK11 + Metal-treated plants), T8 (LHL10 + LK11 + Metal-treated plants). Different letters indicate significant differences between means at *p* > 0.05 (DMRT). Values represent means (of four replication) ± standard error.

In the current study, different key genes (*GmHMA13, GmHMA18, GmHMA19, GmPHA1*, and *GmARI1*) modulating the heavy-metal stress responses and efflux transporters were assessed in the roots and leaves of *G. max* plants co-inoculated with endophytic LHL10 and LK11 under the combined Al and Zn stress conditions (**Figure 2**). The qPCR data revealed that the relative expressions of *GmHMA13* and *GmHMA19* were significantly upregulated in the co-inoculated roots and leaves under no-stress condition as compared to the sole-inoculated and non-inoculated plants. The expression of *GmHMA18* was significantly upregulated in the roots and leaves of the sole LK11-inoculated plants followed by the LHL10-inoculated, dual-inoculated, and non-inoculated plants. The roots and shoots of the non-inoculated plants showed significantly higher *GmPHA1* expression than those of the co-inoculated and sole inoculated plants under no stress condition. However, under stress conditions, the combined toxicity of Al and Zn significantly up-regulated the transcript levels of *GmHMA13, GmHMA18*, and *GmHMA19* in the non-inoculated plant roots and leaves as compared to the co-inoculated and sole-inoculated plant roots and leaves. The endophytic co-inoculation significantly down-regulated the *GmHMA13, GmHMA18*, and *GmHMA19* expression by (12.5, 7.51, and 2.05), (8.48, 1.38, and 3.38) and (56.4, 14.6, and 6.25) fold in roots and (9.54, 5.42, and 1.80), (8.51, 1.77, and 2.51) and (25.4, 10.7, and 2.6) fold in shoots, compared with the non-inoculated, LHL10-inoculated, and LK11-inoculated plants, respectively, under heavy metal stress. Similarly, the relative expression of the plasma membrane H^+^-ATPase gene *GmPHA1* and ariadne-like ubiquitin ligase gene *GmARI1* responsible for soybean tolerance to Al toxicity and detoxification showed that co-inoculation significantly upregulated the transcript level of *GmARI1* as compared to the non-inoculated and sole LHL10- and sole LK11-inoculated plant roots and leaves, but did not show significant difference in the relative expression of *GmPHA1* in comparison with sole LHL10- and sole LK11-inoculated plant roots and leaves under no stress condition. Furthermore, co-inoculation under metal stress significantly downregulated the *GmPHA1* expression in soybean roots by (9.4, 1.4, and 1.9) times and (4.9, 1.2, and 1.8) times in shoots as compared to the non-inoculated, sole LHL10-inoculated, and sole LK11-inoculated soybean, respectively. However, the transcript levels of *GmARI1* were significantly upregulated in the roots of the co-inoculated soybean, exhibiting (7.6, 1.6, and 2.0) times and (3.7, 1.5, and 1.7) times escalation in leaves compared to the non-inoculated, sole LHL10-inoculated, and sole LK11-inoculated soybean roots and shoots, respectively.

### Effect of Co-inoculation on Soybean Macronutrients

**FIGURE 3:**
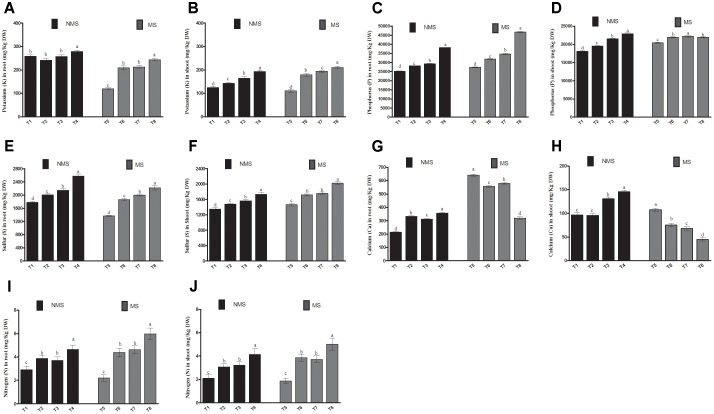
Influence of endophytic LHL10 and LK11 association on soybean root and shoot macronutrient uptake under metals stress. NMS represent “No metal stress” while MS represents “Metal stress.” T1 (Control Plants), T2 (LHL10-treated plants), T3 (LK11-treated plants), T4 (LHL10+LK11-treated plants), T5 (Control + Metals), T6 (LHL10 + Metal-treated plants), T7 (LK11 + Metal-treated plants), T8 (LHL10 + LK11 + Metal-treated plants). Different letters indicate significant differences between means at *p* > 0.05 (DMRT). Values represent means (of four replication) ± standard error.

To determine the effects of co-inoculation on the macronutrient status of soybean, four nutrients, including K, P, Ca, and S, were analyzed in their roots and shoots (**Figure 3**). Compared with the non-inoculated and sole-LHL10- and sole LK11-inoculated, the co-inoculated plants significantly (*p <* 0.005) regulated root macronutrients by displaying substantial increase in the concentrations of K, P, Ca, and S under control conditions. The similar trend was observed in the root tissues under metal-induced stress, where the application of co-inoculation remarkably enhanced the K, P, and S contents. However, under stress conditions, the non-inoculated plant roots showed significantly higher Ca content, followed by the sole LK11-inoculated, sole LHL10-inoculated, and co-inoculated plants roots, respectively. Similarly, under control conditions, a significant increase was detected in the macronutrients of the shoots tissues of the co-inoculated plants as compared to the sole inoculated and non-inoculated plant shoots. The shoots of the co-inoculated plants showed an increase in K by 1.54, 1.35, and 1.17 times, in P by 1.26, 1.17, and 1.06 times, in Ca by 1.50, 1.52, and 1.11, and in S by 1.28, 1.17, and 1.10 times as compared to the shoots of the non-inoculated, sole LHL10-inoculated, and sole LK11-inoculated plants, respectively. Under stress conditions, the co-inoculated plants showed differential regulation of macronutrients in shoots, as K and S were detected to be significantly enhanced, whereas Ca was significantly reduced in the co-inoculated plant shoots, followed by the sole LHL10-inoculated, sole LK11-inoculated and non-inoculated plants shoots. However, the sole LHL10-inoculated plants exhibited significantly (*p <* 0.005) higher P contents, whereas the non-inoculated plants presented significantly lower P contents under stress conditions. Furthermore, the effect of endophytes co-inoculation on nitrogen assimilation of soybean under control as well as Al/Zn stress revealed significant (*p <* 0.05) assimilation of nitrogen in both roots and shoots compared to sole LHL10-inoculated, sole LK11-inoculated and non-inoculated plants.

### Effect of Co-inoculation on Soil Extra-Cellular Enzymatic Activities Under Al and Zn Stress

The activities of soil enzymes, including glucosidase, phosphatase, glucuronidase, and cellulase, were measured to assess the metal detoxification efficiency of co-inoculation for rehabilitating the quality and efficiency of soil. In the current study, inoculating soil either with sole LHL10 endophyte, sole LK11 endophyte, or with both endophytes significantly regulated soil enzymatic activities both under metal-induced stress and non-stress conditions (**Table 3**). In the absence of metal stress, the phosphatase activity of the endophyte co-inoculated plants significantly increased by 35.42, 23.10, and 85.62% compared to those of the sole LHL10-inoculated, sole LK11-inoculated, and non-inoculated plants, respectively. Likewise, a higher activity trend under no-stress condition was displayed by cellulase in the co-inoculated soil, followed by the sole LK11-inoculated, sole LHL10-inoculated, and non-inoculated soil, respectively. In case of glucuronidase under no-stress condition, the co-inoculated soil exhibited significantly higher activity than the non-inoculated and sole LK11-treated soils, but displayed no significant differences with the LHL10-inoculated soil. The co-inoculated and sole LK11-inoculated soils significantly upregulated the glucosidase activity under no-stress condition compared to the LHL10-inoculated and non-inoculated soils. Under Al and Zn contamination, the co-inoculation treatment significantly (*p <* 0.005) enhanced the phosphatase activity by 1.26, 1.51, and 2.23 times, the cellulase activity by 1.10, 1.51, and 2.02 times, and the glucuronidase activity by 1.23, 1.21, and 1.53 times compared to the sole LHL10-inoculated, sole LK11-inoculated, and non-inoculated plants, respectively. The sole LK11-inoculated and co-inoculated soil exhibited almost similar glucosidase activities, followed by the sole LHL10-inoculated and non-inoculated soils under Al and Zn contamination.

**Table 3:** Effect of LHL10 and LK11 on soil enzymatic activities in Zn and Al contaminated soil.

Treatment	Phosphatase (μM^-1^min^-1^mL)	Cellulase (μM^-1^min^-1^mL)	Glucuronidase (μM^-1^min^-1^mL)	Glucosidase (μM^-1^min^-1^mL)
**Without metals stress**				
Control	197.85 ± 6.57^d^	42.15 ± 2.35^d^	34.67 ± 2.84^b^	30.16 ± 1.08^c^
LHL10	299.74 ± 6.69^b^	56.02 ± 2.09^b^	39.33 ± 1.51^a^	33.05 ± 1.57^b^
LK11	272.34 ± 6.75^c^	48.54 ± 2.12^c^	32.49 ± 1.62^b^	36.01 ± 1.77^a^
LHL10 + LK11	368.57 ± 8.37^a^	68.79 ± 2.77^a^	42.29 ± 1.58^a^	35.25 ± 1.21^ab^
**With metals stress**				
Control + Metals	221.73 ± 7.39^d^	27.47 ± 2.58^d^	25.42 ± 1.05^c^	14.76 ± 0.91^c^
LHL10 + Metals	387.38 ± 7.81^b^	68.39 ± 2.61^b^	31.84 ± 0.93^b^	36.861 ± 1.56^a^
LK11 + Metals	318.94 ± 9.87^c^	50.54 ± 2.65^c^	32.68 ± 1.46^b^	28.292 ± 1.32^b^
LHL10 + LK11 + Metals	489.86 ± 8.53^a^	75.49 ± 2.51^a^	39.65 ± 1.22^a^	38.79 ± 1.13^a^


*Values in the columns indexed by different letters are significantly different at *P* ≤ 0.05 based as evaluated by the DMRT. Values in a column represent the mean ± SD of treatments replicated four times fromthree independent experiments.*

### Modulation of Soybean Antioxidant System by Co-inoculation Under Al/Zn Exposure

**FIGURE 4:**
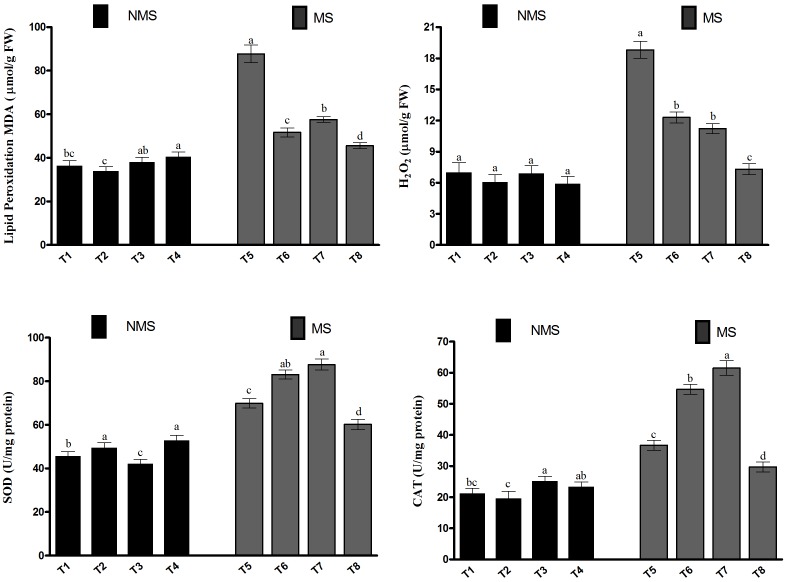
Effects of endophyte inoculation on malondialdehyde (MDA) content, H_2_O_2_ production, and antioxidant activities of soybean under Al and Zn stress. NMS represent “No metal stress” while MS represents “Metal stress.” T1 (Control Plants), T2 (LHL10-treated plants), T3 (LK11-treated plants), T4 (LHL10 + LK11-treated plants), T5 (Control + Metals), T6 (LHL10 + Metal-treated plants), T7 (LK11 + Metal-treated plants), T8 (LHL10 + LK11 + Metal-treated plants). Bars with different letters are significantly different at *p* > 0.05 (DMRT). Values represent means (of four replicates) ± standard error.

To assess the extent of lipid peroxidation in soybean due to metal-induced oxidative stress, the malondialdehyde (MDA) content was investigated (**Figure 4**). The metal-induced stresses generate MDA, which in turn lead to the induction of lipid peroxidation. In the current study, the MDA content in the co-inoculated plant leaf tissues was comparable with that in the sole LK11-inoculated plant leaf tissues and significantly higher than those of the non-inoculated and sole LHL10-inoculated plant leaf tissues (**Figure 4**). However, the Al and Zn stress exposure substantially increased the MDA levels in the non-inoculated plants compared to the co-inoculated and sole inoculated plants. The MDA content of the co-inoculated plant leaves was significantly reduced by 26.1, 13.3, and 92.3% under metal stress conditions relative to the MDA contents of the sole LK11-inoculated, sole LHL10-inoculated, and non-inoculated plant leaves. Similarly, the co-inoculated or sole inoculated plants revealed insignificant changes in the H_2_O_2_ content compared to the non-inoculated plants in the absence of stress; this was also validated by the presence of hydrogen peroxide in soybean leaves using the diaminobenzidine (DAB) staining technique (**Supplementary Figure S1**). However, the Al- and Zn-induced stress significantly triggered the generation of H_2_O_2_ in the non-inoculated plants as compared to the inoculated plants. The co-inoculated plants demonstrated significantly lower H_2_O_2_ content than the sole inoculated and non-inoculated plants, suggesting the alleviation of metal-induced stress. To encounter the metal-induced oxidative stress, the activities of the antioxidant enzymes SOD and CAT were investigated (**Figure 4**). The results depicted that the sole inoculation and co-inoculation treatments differentially regulated the SOD and CAT activities under no-stress control conditions, as both co-inoculated and sole LHL10-inoculated plants significantly (*p <* 0.005) enhanced the SOD activity compared to the non-inoculated and sole LK11-inoculated plants. Under no-stress control conditions, the CAT activity was remarkably higher in the sole LK11-inoculated plants, followed by the co-inoculated, non-inoculated, and sole LHL10-inoculated plants. However, under metal-stress conditions, the sole LK11-inoculated plants presented significantly high CAT activity. However, the co-inoculated plants exhibited 2.06-, 1.83-, and 1.23-fold lesser CAT activity compared to the sole LK11-inoculated, sole LHL10-inoculated, and non-inoculated plants, respectively.

### Influence of Endophyte Co-inoculation on Soybean Endogenous Gibberellic Acid Content

**FIGURE 5:**
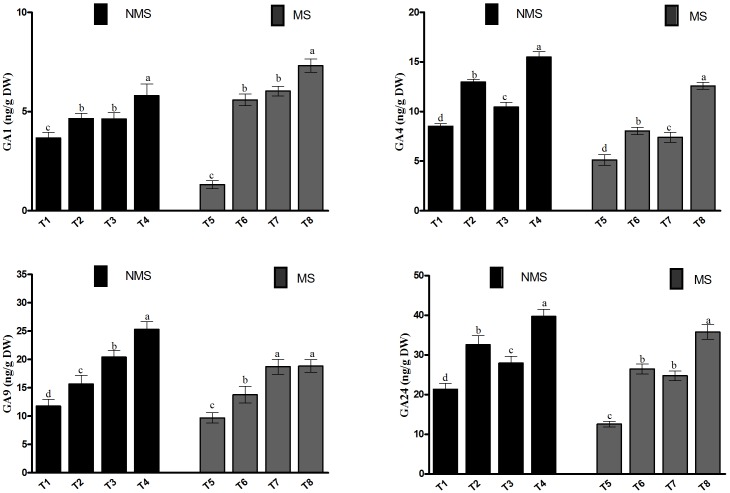
Effect of endophytic LHL10 and LK11 inoculation on the endogenous levels of GAs in soybean under metal stress. NMS represent “No metal stress” while MS represents “Metal stress.” T1 (Control Plants), T2 (LHL10-treated plants), T3 (LK11-treated plants), T4 (LHL10 + LK11-treated plants), T5 (Control + Metals), T6 (LHL10 + Metal-treated plants), T7 (LK11 + Metal-treated plants), T8 (LHL10 + LK11 + Metal-treated plants). Different letters show significant differences between means at *p* > 0.05 (DMRT). Values represent means (of four replication) ± standard error.

In the current study, the endogenous GA (GA_1,_ GA_4,_ GA_9,_ GA_24_) contents of the soybean plant were investigated because of their significance in metal-induced stresses (**Figure 5**). The results depicted that the metal-induced stresses significantly affected the GA contents under metal stresses, particularly in the non-inoculated plants. We found that the co-inoculated plants significantly enhanced the contents of GA_1,_ GA_4,_ GA_9_, and GA_24_ (5.8, 15.4, 25.3, and 39.7 ng/g DW), followed by the sole LHL10-inoculated, sole LK11-inoculated, and non-inoculated plants under no-stress control conditions. Similarly, the sole LHL10- and sole LK11-inoculated plants displayed higher contents of GA_1,_ GA_4,_ GA_9_, and GA_24_ as compared to the non-inoculated plants, but exhibited no significant differences with each other in case of GA_1_ under the control conditions. However, under Al and Zn stress, the co-inoculated plants displayed 5.5-, 1.3-, and 1.2-fold and 2.5-, 1.5-, and 1.6-fold higher contents of GA_1_ and GA_4_ as compared to the non-inoculated, sole LHL10-, and sole LK11-inoculated plants, respectively. Similarly, under stress conditions, the co-inoculated and sole LK11-inoculated plants showed no statistical difference in their GA_9_ contents, but exhibited approximately 37.44 and 94.7% higher GA_9_ production as compared to the sole LHL10-inoculated and non-inoculated plants, respectively. Similarly, the GA_24_ production was recorded significantly higher (35.77 ± 1.8 ng/g DW) in the co-inoculated plants, followed by the sole LHL10inoculated plants (26.4 ± 1.2 ng/g DW), sole LK11-inoculated plants (24.73 ± 1.2 ng/g DW), and non-inoculated plants (12.57 ± 0.72 ng/g DW) under Al and Zn stress.

### Effects of Co-inoculation on Endogenous ABA and JA Modulation of Soybean

**FIGURE 6:**
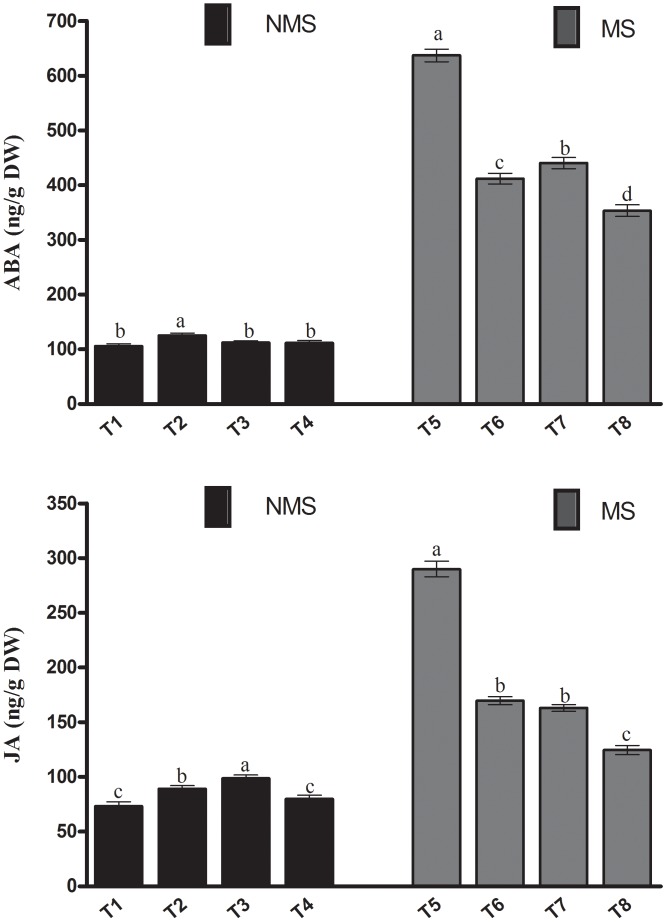
Effect of endophyte inoculation on endogenous abscisic acid (ABA) and jasmonic acid (JA) content of soybean under metal stress. NMS represent “No metal stress” while MS represents “Metal stress.” T1 (Control Plants), T2 (LHL10-treated plants), T3 (LK11-treated plants), T4 (LHL10 + LK11-treated plants), T5 (Control + Metals), T6 (LHL10 + Metal-treated plants), T7 (LK11 + Metal-treated plants), T8 (LHL10 + LK11 + Metal-treated plants). Different letters indicate significant differences between means at *p* > 0.05 (DMRT). Values represent means (of four replication) ± standard error.

The current results depicted that in the absence of metal stress, the stress responsive endogenous ABA levels were not significantly different among the non-inoculated, co-inoculated, and sole LK11-inoculated plants. However, the ABA content of the sole LHL10-inoculated plants significantly increased by 18.1, 11.6, and 12.2% compared to the non-inoculated, sole LK11-inoculated, and co-inoculated plants, respectively (**Figure 6**). Under metal stress, the escalation in the ABA production of plants was noticed among all treatments. However, in comparison with the non-inoculated plants, significantly (*p <* 0.005) lower ABA production was detected in the sole inoculated and co-inoculated plants under stress conditions. Co-inoculation under metal stress significantly reduced the ABA levels by 80.27, 16.59, and 24.62% as compared to the non-inoculated, sole LHL10-inoculated, and sole LK11-inoculated plants, respectively. In case of the endogenous JA content, the sole LK11-inoculated plants significantly (*p <* 0.005) enhanced the production of JA, followed by the sole LHL10-inoculated, co-inoculated, and non-inoculated plants, respectively (**Figure 6**). However, the exposure to metal stress markedly alleviated the JA contents of the non-inoculated plants by more than 2- and approximately 1.7-fold as compared to the co-inoculated and sole-inoculated plants, respectively.

## Discussion

The phytoremediating techniques based on suitable heavy metal-resisting and -tolerating microbes have gained considerable attention in the modern era. Generally, the interactions of endophytes (bacteria and fungi) with crop plants under harsh environmental conditions, including metals stresses, are acknowledged for boosting plant growth and metabolic processes. The individual applications of endophytic fungi, such as *Lasiodiplodia* sp.*, Phialocephala fortinii*, and *Rhizoscyphus* sp., and endophytic bacteria, such as *Pseudomonas* sp., *Methylobacterium oryzae*, and *Sphingomonas* sp., have been reported for increasing plant biomass and ameliorating adverse effects of different heavy metal stresses (Ma et al., 2016a; Yamaji et al., 2016). Some studies have reported the beneficial effects of dual inoculation with arbuscular mycorrhizal fungi, such as *Glomus* sp., *Acaulospora* sp., and *Scutellospora* sp., and plant growth-promoting rhizobacteria, such as *Actinomycetes* sp., *Paenibacillus* sp., *Pseudomonas* sp., and *Azotobacter* sp. on *Pennisetum glaucum*, and *Sorghum bicolor*, which showed a significant increase in plant growth, biomass, and bioremediation of the iron-contaminated soil (Mishra et al., 2016). However, knowledge is lacking on the association of fungal and bacterial endophytes and their interaction with *G. max*, as well as the underlying mechanism for encountering stresses in the metal-contaminated soil. In the current study, a vital growth-benefiting impact of the synergistic consortia of endophytic *Sphingomonas* sp. LK11 and endophytic *Paecilomyces formosus* LHL10 was observed on *G. max* plants under the combined toxicity of Al and Zn. On other hand, a decline in plant growth and biomass was detected in non-inoculated plants, likely resulting from the impairment of the Calvin cycle, photosynthetic machinery, and electron transport chain, as well as from the induction of ROS under combined Al and Zn toxicity.

In the current case of dual inoculation, the potential to produce secondary metabolites, e.g., GAs and IAA, of endophytes might be a contributing factor toward tolerating metal toxicity and promoting growth attributes via enhancing nutrient uptake by proliferating roots and mediating roots exudates under normal as well as Al- and Zn-contaminated soil (Velivelli et al., 2014; Singh et al., 2016). As microbial IAA can loosen the roots cells walls and change the properties of root absorption by increasing root, hairs, length and surface as well as enhancing the excretion of root exudates, which subsequently provide additional nutrients to support the growth of microorganism (Etesami, 2018). Such roots exudates are considered vital for dealing with heavy metals toxicity by preventing them from entering the cell symplast by chelating and forming complexation in rhizosphere; thereby decreasing the metal ion toxicity toward the plant and microbes. Both fungi and bacteria with phytohormones (IAA,GA) producing potential and metals remediating capabilities are repeatedly acknowledged for counteracting heavy metals toxicity (Deng and Cao, 2017; Etesami, 2018). Therefore, mitigating metals toxic effects from in co-inoculated soybean could be ascribed to the cumulative effects of endophytes i.e., metals (Al/Zn) remediating ability and phytohormones producing capability of LHL10 and LK11. Our current findings are also in conformity with the findings of Rajkumar et al. (2012), who extensively reviewed the effects of dual inoculation of mycorrhizal fungi with metals remediating bacteria (*P. putida* or *B. cereus*) and reported their positive influence on host plant growth and heavy metals tolerance.

To explore the alleviation mechanism of metal toxicity-induced stress, it is important to scrutinize the distribution, translocation, immobilization, and availability of metals in the roots, shoots and soil. In the current study, the reduced accumulation of Al/Zn in the roots and shoots of the co-inoculated plants was evident when compared with the non-inoculated and sole inoculated plants. The improved growth of co-inoculated *G. max* L. could possibly be related with the reduced accumulation of Al and Zn in the tissues because of a decrease in their bioavailability in the soil through the absorption/desorption mechanism or preventing their entrance into the tissues by the intracellular accumulation of metals or by the sequestrations of metals in the soil via exudation of various metabolites. Moreover, the initial screening of endophytic LHL10 and LK11 survival against combine Al and Zn toxicity revealed a significant reduction of metals in the liquid broth and exhibiting remarkable uptake via intracellular absorption. Therefore, the reduced metals accumulation and uptake in *G. max.* L tissues could probably be due to the locking down of Al and Zn in their biomass, consequently preventing the plants from their toxic effects. The potential of producing extracellular metabolites, i.e., IAA and gibberellins by endophytic LHL10 and LK11 might have facilitated the metal remediation process and alleviated their toxicity-induced stress in plants. As the production of IAA and GAs by microbes has been reported for boosting plant tolerance to heavy metals toxicity (Deng and Cao, 2017). The deployment of both intra and extra cellular mechanisms simultaneously by LHl10 and LK11 could be a possible justification for the reduction of Al and Zn in the tissues of the endophytic LHL10 and LK11-co-inoculated *G. max* plants. Additionally, the genomic analysis of LK11 (unpublished data) revealed that the LK11 plasmid and chromosome harbor a metal-resisting and -degrading operon containing an active efflux system for accumulated metals; this can be correlated with the reduced metal uptake in plants by assisting the Al and Zn detoxification process in soil. The significantly reduced Al and Zn accumulation and their translocation from roots to shoots of the co-inoculated plants in the current study are in concordance with Fernández-Fuego et al. (2017) and Zahoor et al. (2017), who found relatively low metal uptake in the endophytic *Mucor* sp. MHR-7-inoculated *Brassica campestris* L. and ectomycorrhizal fungus *Paxillus ammoniavirescens*-inoculated *Betula pubescens* Ehr. Contrary to the decrease in metal uptake in the current study, Yihui et al. (2017) found a substantial uptake of Pb in the endophytic *Gaeumannomyces cylindrosporus*-infected *Zea mays* L. Despite the above explanations for reduce metal uptake in *G. max*, the influence of the endophytic fungal LHL10 and endophytic bacterial LK11 co-inoculation on plant Al and Zn cannot be generalized. The uptake and translocation of metals in plants tissues is also reported to be reliant on the interaction between the nature of plants and bacteria/fungi and is also associated with the levels of toxic heavy metals, macro and micronutrients, and soil pH, which eventually critically influence the bioavailability of metals in soil and alter their uptake in plants (Islam et al., 2016; Fernández-Fuego et al., 2017). However, the current findings that indicate the inoculation of the consortia of endophytic bacteria and fungi as a viable strategy for bioremediating the Al- and Zn-contaminated soil and reducing metal uptake in soybean.

In the current study, the plasma membrane H^+^-ATPases genes, which are responsible for providing metal resistance, absorption, and transportation in plants, were investigated. Current findings of the study displayed that the heavy metal ATPase gene *GmPHA1*, which is involved in Al accumulation and translocation from the root to shoot of soybean was significantly downregulated in the roots and leaves of the LHL10- and LK11-co-inoculated plants. Such low expression could be correlated with Al accumulation and uptake, which was significantly reduced by the dual inoculation of endophytes in soybean roots and shoots. Similarly, we investigated to explore the gene expression profiles of P_1B_-ATPases, which are known for transporting diverse range of metals across biological membranes. Therefore, the expression responses of Zn^2+^-ATPases genes (*GmHMA13, GmHMA18*, and *GmHMA19*), which is a subclass P_1B_-ATPases, was assessed to analyze the influence of endophyte dual inoculation on Zn uptake and transportation in soybean. Results revealed that the expression levels of *GmHMA13, GmHMA18*, and *GmHMA19* were remarkably downregulated under Zn stress in the dual-inoculated plants root and leaf tissues compared to other treatments. Such downregulation of genes suggest a significant reduction of Zn accumulation and transportation. Interestingly, the ICP-MS findings of the current study evidenced a remarkable decrease of metals in the dual-inoculated soybeans tissues, which might have contributed to the amelioration of adverse effects of excessive metal accumulation in plants tissues. Recent studies have also discovered the vital roles of ariadne-like E3 ubiquitin ligases in plant responses to abiotic stresses, including metal toxicity (Lang-Mladek et al., 2012; Zhang et al., 2014). Therefore, the evaluation of ariadne-like E3 ubiquitin ligase gene *GmARI1* showed that dual inoculation modulated the *GmARI1* gene by revealing significant expression under Al stress conditions. The upregulation of *GmARI1* has been reported for enhancing soybean tolerance and resistance to severe Al toxicity by regulating plant response to Al through suppressing the oxidative stress signals, which could interact with plant hormone signaling pathways to reduce the metal-induced damage or root growth inhibition in plants (Zhang et al., 2014; Gao et al., 2017). The increased transcript accumulation of *GmARI1* in the roots tissues compared to the leaf tissues could be related to the fact that Al stress initially causes damage to plant roots and subsequently leads to uptake of water and nutrient inhibition, thereby affecting plant growth and development. The increased transcript accumulation of *GmARI1* in co-inoculated plants might be attributed to the combined exogenous production of IAA by endophytic LHL10 and LK11 under Al stress, as the exogenous application of IAA significantly upregulated the *GmARI1* expression under Al contamination in soybean roots (Zhang et al., 2014). In current study such decreased plasma membrane H^+^-ATPases transporters activity of co-inoculated supports the fact that consortia of endophytic LHL10 and LK11 significantly mitigated Al/Zn toxic effects in soybean by reducing their uptake and accumulation, which could be an effective strategy for reducing metal transfer from the contaminated soil to the food chain.

In addition to adverse impacts on plant growth and biomass, the toxicity of metals, including Al and Zn, negatively influences the availability and uptake of essential elements in plant roots and shoots (Bhattacharya et al., 2010). Conversely, in the current study, the synergistic association of bacterial and fungal endophytes demonstrated a significant increase in the uptake of P, K, and S in plants roots and shoots under metals stress, suggesting that dual inoculation in soybean strictly regulates the uptake and accumulation of both essential and non-essential nutrients. The enhancement in the uptake of P, K, S and N uptake in roots and shoots of the dual-inoculated soybean plants might be due to the nutrient-mobilizing activity and phytohormone (IAA and GAs)-producing potential of the endophytic LK11 and LHL10. The phytohormone production by endophytes associated with the plants under abiotic stresses frequently stimulate the growth and metabolic processes, leading to an extended root system, which effectively contributes to augmented nutrient uptake. As previously endophytic bacteria *Pseudomonas brassicacearum* and endophytic fungi *P*. *fortinii, Rhizodermea veluwensis*, and *Rhizoscyphus* sp. have been reported for enhancing the uptake of host plant macronutrients under diverse metals toxicity conditions (Yamaji et al., 2016; Kong and Glick, 2017). However, in dual inoculated plants, a decrease in Ca uptake was recorded under metal stress compared to other treatments. An increase in the Ca content of the non-inoculated plants compared to the co-inoculated ones could be related to the significant production of H_2_O_2_ under metal-induced toxicity, triggering the activation of hyperpolarization-activated calcium channels (HACCs) for enhancing Ca^2+^ influx to directly stimulate NADPH oxidase for generating ROS, which might lead to the generation of ABA for regulating plant responses to stress mitigation (Kar, 2011; Leitão and Enguita, 2016). Therefore, in the current study, the considerable decrease in H_2_O_2_, Ca accumulation, and ABA production of co-inoculated soybean under metal toxicity could be corroborated with the positive influence of co-inoculation in soybean under combined Al and Zn toxicity.

The sources of soil enzymes have not been fully explored, but the soil microbial community and plants are thought to be the major sources of soil enzymes. Soil extracellular enzymes are considered to be involved in diverse biochemical reactions: reducing metal-induced soil toxicity, maintaining soil texture and structure, conversion and decomposition, cycling and decomposition of organic matter. Numerous studies have demonstrated that heavy metal contamination significantly impairs soil enzymatic potential by interacting with the enzyme-substrate complex and consequently causes the denaturation of enzyme proteins (Ma et al., 2015). The current study revealed that the application of the endophytic LHL10 and LK11 co-inoculation efficiently tackled the lethal effects of combined Al and Zn toxicity by augmenting soil enzymatic activities. Endophyte co-inoculation of plants exponentially improved the phosphatase activity of soil compared to the non-inoculated plants under stress conditions. The enhancement of soil phosphatase activity under metal contamination is the major mechanism recruited for avoiding and detoxifying heavy metal toxicity as well as improving plants tolerance to metal stresses (Wang et al., 2006). Phosphatases are also considered to be involved in the breakdown of various phosphate esters and alternatively enhance phosphorus (P) availability and uptake by plants; this was evidently witnessed in the co-inoculated plants in the current study. Such significant increase demonstrates the positive influence of endophytic co-inoculation in metal detoxification and plant growth enhancement. Curaqueo et al. (2014) and Ali et al. (2017) reported significant enhancement in the phosphatase activity of the soil polluted with various heavy metals by inoculating it with AM fungi and *Streptomyces pactum* (Act12). Similarly, a significant improvement was detected in the cellulase activity of the dual inoculated soil under combined Al and Zn contamination. Jia et al. (2015) and Xian et al. (2015) reported that the heavy metal contamination of soil lead to a reduction in its cellulase activity. An escalation in the cellulase activity of the dual-inoculated soil increases the breakdown of carbohydrates and decomposition of organic matter and is considered crucial for assisting the vertical transmission and colonization of endophytes in plants (Ma et al., 2011; Xian et al., 2015). Likewise, Burges et al. (2015) and Parelho et al. (2016) found that the glucosidase activity of an agricultural soil is highly sensitive to metal toxicity and is significantly reduced. On the contrary, the endophytic dual inoculation under combined Al/Zn toxicity significantly maintained the glucosidase activity similar to that in the control conditions. This suggests that the synergistic inoculation of endophytic LHL10 and LK11 as a potential strategy for supporting soil microbial community and offering a friendly ecosystem for eventual plant growth and yield enhancement under Al- and Zn-contaminated soil conditions.

The current study revealed that the accumulation and translocation of the reducing metals (Al and Zn) in the *G. max* tissues led to a considerable mitigation of the metal-induced oxidative stress compared to the sole inoculation and non-inoculation. The alleviation of Al- and Zn-induced oxidative stress could also be related to the direct impact of dual inoculation on plant biomass, leading to the development of both underground and aboveground plant parts. Excessive accumulations of Al and Zn in plants result in significant inhibition of the photosynthetic electron transport chains, which ultimately lead to the generation of ROS, such as superoxide anion, H_2_O_2_, and singlet oxygen. Therefore, the dual inoculation of plants considerably influenced their metal uptake in the current study, thereby reducing the level of lipid peroxidation of membranes by exhibiting nearly same amount of MDA as in the absence of metal stress. Similarly, the excessive generation of H_2_O_2_ due to excessive metal accumulation results in the oxidation of cysteine and methionine residues and tempers the Calvin cycle enzymes and photosynthetic system (Das and Roychoudhury, 2014). However, the inoculation of multiple endophytes under combined Al and Zn toxicity displayed lower production of H_2_O_2_, suggesting their Al and Zn stress-mitigating effect. The improved chlorophyll content of the co-inoculated plants under metal stress condition could also be attributed to the significantly lower production of H_2_O_2_ and MDA. The membrane lipids are extremely vulnerable to the oxidation of metal-induced ROS and considered as the first target of metal toxicity. However, plants generally deploy the synergistic activation of antioxidants to counteract the overproduction of metal-induced ROS. Therefore, as major defense enzyme against metal-induced oxidation, SOD acts as the first line of defense for counteracting metal toxicity by dismutating super oxide radicals into H_2_O_2_ and O_2_ via Haber-Weiss reaction. Moreover, the deployment of tetrameric heme-containing CAT further carries out the dismutation of H_2_O_2_ into H_2_O and O_2_ (Sharma et al., 2012). However, in the current study, endophyte dual inoculation resulted in the down regulation of the SOD and CAT activities of *G. max* grown under Al- and Zn-contaminated soil. Such low CAT and SOD activities of co inoculated treatment as compared to non-inoculated and sole inoculated treatments could be the consequences of the endophytic LHL10 and LK11 co-inoculation-induced inhibition of metal entrance and subsequent ROS production in plants. Contrary to the dual inoculation, the sole inoculation led to the significant enhancement of the SOD and CAT activities of plants under metal contamination compared to the non-inoculated plants. However, the possible explanation for such variations in the antioxidative activities of the sole- and dual-inoculated plants could be that the combined metal sequestration/immobilization effect of LHL10 and LK11 makes Al and Zn less accessible to plants, lowering down their uptake. Consequently, host plants are successfully escaped from the metal-induced ROS generation, thereby inhibiting the generation of SOD and CAT. The reduced generation of ROS, i.e., MDA (lipid peroxidation), H_2_O_2_, SOD, and CAT antioxidative enzymes suggests a promising role of endophytic dual inoculation in successfully alleviating metal stress in soybean.

Plant endogenous hormones play a crucial role in plant growth and development, and their interplay is of great significance to better understand the stress-coping mechanisms of plants. There are strong evidences that phytohormones (ABA, JA and GA) play vital role in the expression of Al and Zn toxicity and the subsequent responses of the plants to their toxicity. Kopittke (2016) extensively reviewed that endogenous hormonal level of plant tissues remarkably change upon exposure to Al stress, which might be either due to direct response of stress responsive hormones to Al stress in order counteract the toxic effects of Al, or due to non-specific change in plant functioning as a result of Al stress. However, the fluctuations of endogenous hormones under hostile conditions including Al/Zn toxicity help to alter plant cellular dynamics and thus play a central role in coordinately regulating growth responses to cope with stresses (Kopittke, 2016; Jalmi et al., 2018). The convergence points among defense responsive hormones signal transduction cascades are referred as crosstalk, and by this way hormones possibly interact together to constitute signal defense networking against environmental stresses.

Limited information is available on the soybean endogenous phytohormone interaction in context to the dual application of endophytes under combined Al and Zn toxicity. The current study revealed the influence of the inoculation of endophytic consortia on endogenous GAs, ABA, and JA production of *G. max* under combined Al and Zn toxicity. GAs are widely recognized for plant growth and development under normal and stress conditions, while their deficiency leads to the susceptibility of plants to various stresses, including metal stress (Asgher et al., 2015). The co-application of endophytic LHL10 and LK11 seems to significantly enhance the production of both bioactive GA_1_ and GA_4_ under control as well as stress conditions. This suggests that the endophytic consortium triggered both the C-13 hydroxylation and non-C-13 hydroxylation pathways for gibberellin biosynthesis under metal stress. The high amounts of GA_1_, GA_4_, GA_9_, and GA_24_ in endophytic co-inoculation treatment suggest that the endophytic consortium significantly maintained plant growth under stress conditions. Additionally, the relatively higher concentration of the bioactive GA_4_ compared to GA_1_ suggests non-C-13 hydroxylation as the major GA biosynthetic pathway in dual-inoculated soybean plants. Our results are in the agreement with the previous findings of Kim et al. (2015) and Hamayun et al. (2017), who reported higher production of GA_4_ compared to GA_1_ and suggested the non-C-13 hydroxylation pathway as the major GA biosynthesis mechanism for growth and development in soybean plant. Endogenous ABA is a multifunctional hormone, whose increased accumulation is considered to be involved in protecting plants against heavy metal stresses including (Al and Zn) via causing stomatal closure for preventing water loss and stress damage. Wani et al. (2016) reported that endogenous level of ABA increase rapidly under environmental stresses including heavy metals stress for activating specific signaling pathways and stress responsible genes expression. As previously Hou et al. (2010) reported significantly accumulation of endogenous ABA level in soybean under aluminum stress. However, in the current study, we detected a significant reduction in the ABA content of co-inoculated soybean under metal stress compared to other treatments and also observed an antagonism between GA and ABA production. As the higher production of ABA in plants is involved in conferring tolerance to various abiotic stresses, but the current reduction in ABA content and enhancement in GAs in metal-affected soybean associated with endophytic dual inoculation demonstrates the alleviation of the combined Al and Zn stress, probably by improving plant growth by the phytohormone (GA, IAA)-producing ability of endophytes and consequently obstructing the accumulation of DELLA proteins. In agreement with this, Al and Zn toxicity also enhanced JA accumulation, showing the possible contribution of JA in stress-mediated signaling. JA has been demonstrated as a critical signaling molecule for boosting plant performance under hostile conditions, including metals stresses (Per et al., 2018). As Huang et al. (2017) reported that transcriptomic analysis of roots and leaves of soybean reveled the significant expression levels of genes involved in jasmonic acid mediated signaling pathway under Al stress. However, it is worth-mentioning that the combined application of endophytes significantly reduced JA levels in soybean under Al/Zn toxicity, suggesting that the application of endophytic consortia has alleviated Al/Zn induced stress in soybean and subsequently lower production of JA was detected. Such reduction of JA in co-inoculated soybean under Al and Zn stress might be ascribed to the enhanced levels of bioactive GAs, leading to the degradation of DELLA proteins and consequently setting the Jasmonate ZIM-domain (JAZs) proteins free to bind with MYC2 and suppressing its activity to attenuate JA signaling (Per et al., 2018). Therefore, a significant increase in the contents GAs and stress-responsive hormones ABA and JA in co-inoculated soybean indicate that the association of LHL10 and LK11 has a beneficial role in mitigating metal stress and promoting plant growth and development.

## Conclusion

The findings of the current study disclosed that the association of the phytohormone-producing endophytic *Paecilomyces formosus* LHL10 and *Sphingomonas* sp. LK11 are coordinately involved in soybean growth promotion and adaptation to combine Al and Zn induced stress. The endophytic dual inoculation improved plant growth via combined mechanisms of lowering metal toxicity in soybean tissues by inhibiting the uptake and translocation of metals and by enhancing the uptake of essential nutrients and modulation of soil extracellular enzymatic activities. This strategy further resulted in the reduction of metal-induced oxidative stress and tight regulation of stress responsive hormones. These observations indicate the application of a consortium of endophytic bacteria and fungi- as a promising technique for ensuring plant growth and safer food consumption under metal-contaminated soil conditions. However, the comprehensive understanding of the mechanistic aspects of the interaction between endophytic bacteria and fungi for improving plant growth and adaptability to metal contamination requires an in-depth study at the proteomic and transcriptomic levels.

## Author Contributions

SB designed the experiments. RS and SB performed the experiments. S-MK, B-WY, QMI, and AK analyzed the data. AK, I-JL, and AA-H edited the manuscript.

## Conflict of Interest Statement

The authors declare that the research was conducted in the absence of any commercial or financial relationships that could be construed as a potential conflict of interest.
