# Supplementary material for: Endophytic Microbial Consortia of Phytohormones-Producing Fungus Paecilomyces formosus LHL10 and Bacteria Sphingomonas sp. LK11 to Glycine max L. Regulates Physio-hormonal Changes to Attenuate Aluminum and Zinc Stresses
Source: Front Plant Sci. 2018 Sep 4;9:1273. doi: 10.3389/fpls.2018.01273 (PMC6131895; doi:10.3389/fpls.2018.01273)
Supplement: Supplementary file 1 [file Data_Sheet_1.docx]

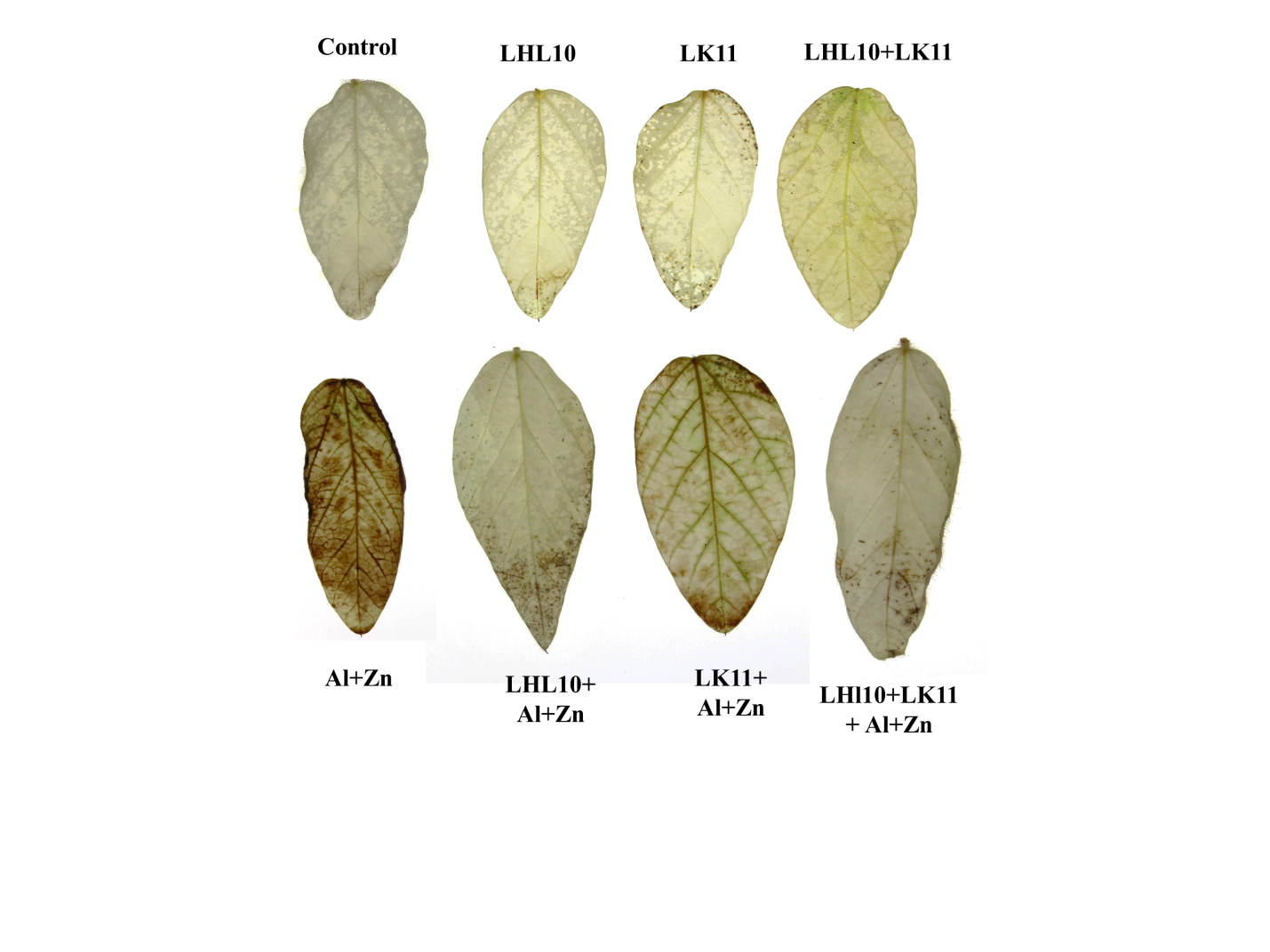


Supplementary Figure 1: Detection of hydrogen peroxide due metals toxicity in Soybean leaves using diaminobenzidine (DAB) staining.
