# Supplementary material for: Endophytic Microbial Consortia of Phytohormones-Producing Fungus Paecilomyces formosus LHL10 and Bacteria Sphingomonas sp. LK11 to Glycine max L. Regulates Physio-hormonal Changes to Attenuate Aluminum and Zinc Stresses
Source: Front Plant Sci. 2018 Sep 4;9:1273. doi: 10.3389/fpls.2018.01273 (PMC6131895; doi:10.3389/fpls.2018.01273)
Supplement: Supplementary file 2 [file Table_1.docx]

**Table S1: List of primers**

| *Genes* | *Primers* |
| --- | --- |
| *GmHMA13* | *Forward: TCGCACGCAATGTCAAAGCC*  *Reverse: CCTCCAATGCCCCTGATCTGC* |
| *GmHMA18* | *Forward: TCAAATTGTTAAGGCACTG*  *Reverse: GGATAGACAGCAACAGCA* |
| *GmHMA19* | *Forward: TCACCACTCCCACGGTGCTA*  *Reverse: GAGTGGCTTGGGCAAGGTGT* |
| *GmPHA1* | *Forward: GAGTGGGAAGGCTTGGGATA*  *Reverse: GTTGGTTGTTTCAGGTGGC* |
| *GmARI1* | *forward: AGTGGTGTCCTGCTCCAGGT*  *reverse: TTGCCACAGTGCCACAGTCC* |

**Table S2. GC/MS – SIM conditions used for analysis and quantification of the GAs**

| Equipment | Hewlett-Packard 6890, 5973N Mass Selective Detector |
| --- | --- |
| Column | HP-1 capillary column (30m×0.25mm i.d. 0.25 µm film thickness) (J & W Scientific Co., Folsom, CA, USA) |
| Carrier gas | He (40 ml/min.); head pressure of 30 kPa |
| Source temperature | 250°C |
| Oven conditions | 60°C (1 min.) → 15°C /min. → 200°C (1 min.) → 5°C /min. → 285°C (5 min.) |
| Injector temperature | 200°C |
| Ionizing voltage  m/z | 70 ev  GA_1_(506, 508), GA_4_(284, 286), GA_9_((298,300), GA_24_(314,316) |

**Table S3. GC/MS – SIM conditions used for analysis and quantification of the ABA**

| Equipment | Hewlett-Packard 6890, 5973N Mass Selective Detector |
| --- | --- |
| Column | HP-1 capillary column (30m×0.25mm i.d. 0.25µm film thickness) (J & W Scientific Co., Folsom, CA, USA) |
| Carrier gas | He (40 ml/min.); head pressure of 30 kPa |
| Source temp. | 250°C |
| Oven conditions | ABA : 60°C (1min.) → 15°C/min. → 200°C →5°C/min. → 250°C →10°C /min → 280°C |
| Injector temp. | 200°C |
| Ionizing voltage  *m/z* | 70 ev  190, 194 |

**Table S4. GC/MS – SIM conditions used for analysis and quantification of the JA**

| Equipment | Hewlett-Packard 6890, 5973N Mass Selective Detector |
| --- | --- |
| Column | HP-1 capillary column (30m×0.25mm i.d. 0.25µm film thickness) (J & W Scientific Co., Folsom, CA, USA) |
| Carrier gas | He (40 ml/min.); head pressure of 30 kPa |
| Source temp. | 250°C |
| Oven conditions | JA : 60°C (2 min.) → 10°C /min. → 140°C (3min) →3°C /min 170°C → 15°C /min. → 285°C (8 min.) |
| Injector temp. | 200°C |
| Ionizing voltage  *m/z* | 70 ev  83, 151, 153 |
